# Real time machine learning prediction of next generation sequencing test results in live clinical settings

**DOI:** 10.1038/s41746-025-01816-7

**Published:** 2025-08-19

**Authors:** Grace Y. E. Kim, Matthew Schwede, Conor K. Corbin, Sajjad Fouladvand, Rondeep Brar, David Iberri, William Shomali, Jean S. Oak, Dita Gratzinger, Henning Stehr, Jonathan H. Chen

**Affiliations:** 1 Stanford Center for Biomedical Informatics Research, Stanford, CA USA; 2 Department of Biomedical Data Science, Stanford, CA USA; 3 Department of Hematology, Stanford, CA USA; 4 Department of Pathology, Stanford, CA USA; 5 Division of Hospital Medicine, Stanford, CA USA; 6 Clinical Excellence Research Center, Stanford, CA USA

**Keywords:** Translational research, Biomedical engineering

## Abstract

Next-generation sequencing-based tests have advanced the field of medical diagnostics, but their novelty and cost can lead to uncertainty in clinical deployment. The Heme-STAMP is one such assay that tracks mutations in genes implicated in hematolymphoid neoplasms. Rather than limiting its clinical usage or imposing rule-based criteria, we propose leveraging machine learning to guide clinical decision-making on whether this test should be ordered. We trained a machine learning model to predict the outcome of Heme-STAMP testing using 3472 orders placed between May 2018 and September 2021 from an academic medical center and demonstrated how to integrate a custom machine learning model into a live clinical environment to obtain real-time model and physician estimates. The model predicted the results of a complex next-generation sequencing test with discriminatory power comparable to expert hematologists (AUC score: 0.77 [0.66, 0.87], 0.78 [0.68, 0.86] respectively) and with the capacity to improve the calibration of human estimates.

## Introduction

**Fig. 1:**
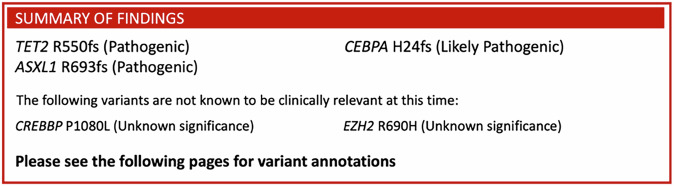
Sample Heme-STAMP report. Sample results from a Heme-STAMP report for a patient with acute myeloid leukemia. Additional elements of the multi-page report include a detailed description of the consequences of each mutation.

Next-generation sequencing (NGS)-based testing is a crucial tool for the care of patients with hematolymphoid neoplasms (blood and lymph cancers). It is used in diagnosis and residual disease testing^1^, personalizing therapy^2^, and for the classification of these diseases^3^. Despite the utility of these diagnostic tests, their cost and novelty can limit clinical deployment. Heme-STAMP (Stanford Actionable Mutation Panel for Hematopoietic and Lymphoid Malignancies) is one such NGS-based genome profiling assay that tracks mutations and fusions in over 200 genes that are implicated in hematolymphoid neoplasms. Hematologists and oncologists use the test for diagnosis and classification of hematologic malignancies^4^, as well as to identify targetable mutations and track disease progression. Figure 1 shows a portion of a Heme-STAMP result for a patient with acute myeloid leukemia (AML), in which the *ASXL1* mutation indicates a worse outcome^5^.

Despite the increasing uptake of such diagnostic tools, there is limited practical guidance for clinicians on when the tests are likely to be clinically useful. Guidelines for NGS use have been published for AML^5^, but inconsistent practice patterns have emerged, with some oncologists under-utilizing this technology^6^, and others potentially over-applying it in unproven clinical scenarios, such as screening for mutations in any patient with abnormal blood counts. Settings where the indication for testing is ambiguous often lack data evaluating NGS test characteristics. Furthermore, the utilization of such diagnostic tools is only increasing among a broad range of physicians of differing specialties, such as cardiology^7^. These trends highlight the growing importance of clinical decision support tools to guide effective test usage and decision-making on whether to order these tests.

Machine learning (ML) model-based clinical decision support tools can help by predicting test outcomes, a critical step in decision-making. While the utility of certain test outcomes depends on context, estimating pre-test probabilities informs whether testing is necessary to discover those outcomes. This principle is rooted in decision analysis^8^; if the pre-test probability is below or above defined thresholds, testing may be unnecessary, as the outcome is already clear. Cases falling between these thresholds benefit most from testing. Since test utility varies by clinical context, we collaborated closely with front-line clinicians (M.S., R.B., D.I., W.S., J.S.O., D.G., and J.H.C.) to design a system tailored for this NGS test. Generating accurate pre-test probabilities is a complex but crucial step in decision-making—one well-suited for ML-based approaches due to its quantitative nature.

In previous work, we demonstrated the potential of an ML model-based approach to predict the results of Heme-STAMP tests based on retrospective data^9^, indicating the potential for personalized pre-test guidance similar to other diagnostics^10^. To better assess clinical impact in patient care settings, a prospective evaluation of real-time predictions and comparison to human expert estimation is needed.

The objectives of this study are (1) to determine how accurately an automated ML model predicts the results of an NGS test in real-time when integrated into a live electronic health record (EHR) environment, and (2) to determine how model-based predictions compare to the physicians who order the tests. Secondary objectives are to assess the combination of the model with human performance and to identify opportunities and barriers for integrating ML-driven diagnostic test decision support systems into clinical practice.

## Results

### Cohort summary

Supplementary Table 3 reports summary statistics of Heme-STAMP cases in the retrospective (train/validation) dataset used for model training and in the prospective (test) dataset. The most common cancers seen in the prospective cohort are among the most prevalent hematologic diseases, such as myeloproliferative neoplasms (Supplementary Fig. 3)^11^.

### Machine learning model has comparable performance to expert hematologists

**Fig. 2:**
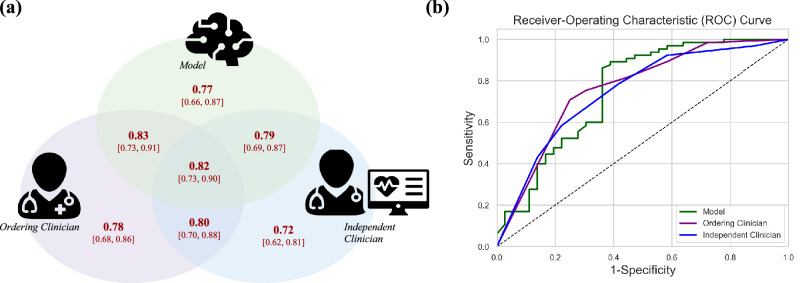
Predictive performance of study arms and ensemble approaches. **a** AUROC scores [95% CI] of predictions made by the model, ordering clinician, independent clinician, or ensemble combination of those three approaches on the prospectively collected set of cases. **b** ROC curves of model, ordering clinician, and independent clinician estimators.

Figure 2 and Supplementary Table 4 shows the predictive performance of each of the three study arms. Model performance was comparable to both ordering and independent clinician estimates on the prospective cohort with AUROC scores of 0.77 [0.66, 0.87], 0.78 [0.68, 0.86], and 0.72 [0.62, 0.81] and AP scores of 0.84 [0.74, 0.93], 0.83 [0.73, 0.91], and 0.80 [0.69, 0.90], respectively. The AUROC score of the model on the prospective cohort was comparable to its performance on the retrospective cohort (AUROC score: 0.76 [0.73, 0.79]).

### Combining physician and model estimates maintains discriminatory power while potentially improving calibration

**Fig. 3:**
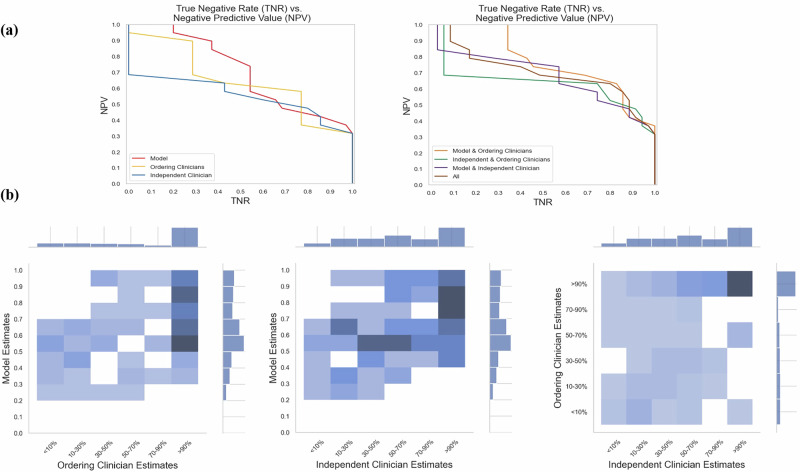
Negative precision-recall curve and heatmap of estimators. **a** NPV-TNR (i.e., negative precision-recall) curves to summarize the prediction performance of the independent predictors (left) and ensemble predictors (right). **b** Distribution of estimates and heatmap with counts for each pair of independent predictors.

We combined the model and physician estimators to create ensemble predictions and found that the AUROC scores of this ensemble approach were comparable to those of the individual predictors (Supplementary Table 4, Fig. 2) and at fixed NPV values of 0.90 and 0.95, the ML model + ordering clinician ensemble had the highest TNR (Fig. 3a).

The physician and model had directional agreement (using 50% threshold) on over 70% of the cases. For the cases where there was disagreement, physician estimates were gathered from a separate set of hematologists before and after showing the model estimates. Despite the differing estimates of the model, physicians only changed their predicted probability value on 20% of discrepant cases, and these predicted probability value adjustments did not change the overall label from what the physician had originally predicted (using a 50% threshold). However, based on the Brier scores, averaging the ordering physician and model estimates (Brier score [95% CI]: 0.21 [0.09, 0.35]) seems to lead to improved calibration (while maintaining discriminatory power) than either the average of the ordering physician and the additional physician queried as a “second opinion” (Brier score [95% CI]: 0.26 [0.13, 0.40]) or the ordering physician estimate alone (Brier score [95% CI]: 0.36 [0.25, 0.50]). We see that the average of the ordering physician and model queried as a “second opinion” had the lowest Brier score and thus best calibration. Due to the overlap between 95% confidence intervals, these findings are suggestive rather than statistically significant.

### Additional analyses

The model shared a statistically significant positive ranked correlation with both groups of clinicians (Supplementary Table 5). This correlation was lower than that between the independent and ordering clinicians, suggesting that the model makes distinct use of the data, rather than simply duplicating a physician’s approach. Clinicians had more skewed estimation distributions than the model, particularly the ordering clinicians, whose predictions were heavily skewed towards >90% (Fig. 3b, Supplementary Fig. 4).

Supplementary Table 4 shows the predictive performance on exploratory subsets (by sample type and age) of the prospective dataset. The model’s and clinicians’ predictive performance were similar across bone marrow and peripheral blood sample types, which is consistent with the known concordance between peripheral blood and bone marrow NGS testing for known or suspected myeloid malignancy^12^. By contrast, the predictive capability of both the model and clinicians tended to be lower for the older half of the cohort (≥67 years old, positive prevalence 0.5) compared to the younger half (<67 years old, positive prevalence 0.5) (Supplementary Fig. 5).

We used SHAP values to identify features most important for prediction. In Supplementary Fig. 6, we can see that the top contributors were important clinical factors often used by hematologists, such as patient age, prior hematologic diagnoses, and elements of the complete blood count. For example, older patients are likely to be predicted to have hematologic mutations, which is expected because of increased clonal hematopoiesis and hematologic disease in older individuals^13^. Similarly, a high white blood cell count increases the likelihood that the presence of a hematologic mutation will be predicted, which is expected given that many hematolymphoid malignancies result in high white blood cell counts.

Lastly, to understand the minority of cases that had discrepancies in estimates between the model and clinicians, the independent clinician manually reviewed a sample of cases where the absolute difference in predicted probabilities between the model and either the ordering or independent clinician was 0.5 or greater (Supplementary Table 6). While some of the discrepancies were due to physician judgment, others were due to the model lacking access to outside records and unstructured text, which contained prior mutation testing information.

## Discussion

We used an ML model based on structured data from the EHR to predict whether next-generation sequencing testing would result in a pathogenic mutation. In prior work^9^, we showed the potential of ML models on this prediction task through retrospective evaluation. Here we deploy the model in a live health system to generate prospective estimates and demonstrate the model’s ability to maintain high performance despite the challenges that come with deployment in a live, production environment.

The first key finding of our study is that the machine learning model has comparable performance to expert hematologists despite having access to less information. We found that the model had comparable performance to both ordering and independent hematologists with AUROC scores of 0.77 [0.66, 0.87], 0.78 [0.68, 0.86], and 0.72 [0.62, 0.81], respectively (Fig. 2, Supplementary Table 4). At fixed negative predictive values of 0.9 and 0.95, the model had the highest true negative rate (specificity), indicating that the model is able to anticipate more negative cases than human experts while maintaining high accuracy. The comparable performance between the model and hematologists is particularly notable, as the model only had access to structured EHR data. In contrast, hematologists had direct interactions with patients and access to unstructured patient data, including clinic notes, external clinic referral information, pathology reports, and imaging results.

In addition, we evaluated the predictions of hematology specialists who were specifically selected based on their experience and regular use of this diagnostic test. In practice, though, an increasingly broad variety of physicians are ordering these tests with different levels of familiarity, expertise, and patient disease prevalence, such as cardiologists looking for mutations associated with cardiovascular disease^7^. This makes the applications of such a machine learning model that can demonstrably perform comparably to expert hematologists even more exciting.

Computational assistance may also be increasingly important as temporal changes are incorporated into clinical decision-making, such as the need for a sustained abnormality to diagnose a blood cancer^3^. With increasing volumes of data, the development of physician decision-support tools will continue to become more critical.

The second key finding of our study is that combining physician and model estimates preserves discriminatory power while improving calibration. While our primary objective was to compare model vs human performance, we recognize that machine-based predictions should complement, not replace, clinical judgment, and so we incorporated secondary analyses of the combination of human and machine predictions. We found that integrating physician and model estimates maintained the same high level of discrimination as physicians alone (Fig. 2) while potentially enhancing calibration. Brier scores indicate that this combined approach achieves better calibration than physicians alone or when averaged with an expert second opinion. Although the confidence interval overlap prevents statistical significance, the finding is highly suggestive and warrants further investigation in a study designed to detect this difference. Calibration—often overlooked in clinical ML analysis—is crucial, as it measures how closely predicted probabilities align with actual outcomes. Unlike discrimination, which ranks risk levels, calibration provides clinicians with more precise probabilities that influence decision-making. For example, a predicted probability of 0.55 versus 0.8 may lead to different clinical actions. While our results are only suggestive, they highlight a promising avenue for future research.

The third key finding of our study is the demonstrated feasibility of integrating a custom machine learning model into a real-world clinical environment using modern EMR APIs, alert infrastructure, and cloud computing. Despite the growing popularity of machine learning in clinical environments, many are not able to surpass the implementation gap between piloted clinical ML models and those in clinical care^14^. Implementation is critical to conducting a prospective evaluation of model performance in a live clinical environment (compared to its performance in the retrospective pilot) and to facilitate a smoother transition to becoming a tool integrated into the workflow. A major hurdle in this transition is the technical challenge of integration. This study not only demonstrates that it is feasible to integrate a custom machine learning model developed in a research lab into a real-time clinical environment, but also shares how.

While most piloted clinical models are trained and evaluated on retrospective clinical data stored and maintained in an isolated data repository, a real-time model in clinical use faces multiple challenges. Implementation requires drawing from a real-time data source, which may be difficult to access, and mapping from retrospective data warehouses may be imperfect. Once accessed, the model needs infrastructure to generate immediate feature vectors from this real-time data when an order is placed, and it should be robust to system or workflow changes in a dynamic clinical environment. Many institutions using machine learning in clinical environments have relied on platforms native to their EHR vendor (eg, Epic Nebula) to build these models, used daily refreshes of their clinical data warehouse (eg, Epic Clarity), or developed custom solutions that are specific to the institution’s unique structure and collaborations^14^.

However, outsourcing can be expensive and is inflexible to the customizations needed for each model use case and institution-specific needs. Using daily refreshes of the EMR does not allow for real-time predictions, which is crucial in many applications. And details of custom solutions are often not shared with the public or are too specific to an institution’s unique structure that others have to reinvent those processes.

In this work, we share a reproducible approach that leverages pathways that others can also utilize. We built upon the DEPLOYR framework^14^ and used: FHIR-based Epic API calls to access real-time data from Epic’s transactional database (Epic Chronicles); the mechanism of an EHR alert to trigger downstream custom actions such as model inferencing; and Microsoft Azure’s cloud computing resources to store the model, pull together real-time feature vectors, and generate inferences. We then tested our model for stability against changes from data mapping and system updates. Our dataset, code, and configuration details are publicly available (see Methods, Data Availability, and Code Availability). Using this system enables deployment of a custom machine learning model in a live clinical environment.

Despite careful mapping of features from retrospective data to those of real-time data, we still observed differences in feature distribution after shifting to a production environment (Supplementary Table 3). Additionally, aspects of the EHR were updated during the study, creating additional mapping challenges. For example, a version update to the EHR introduced changes in medication naming, and the Heme-STAMP test underwent an update, expanding the number of genes sequenced. Despite these changes, the model was sufficiently robust and maintained comparable performance between retrospective and prospective cohorts. Such dynamic changes are inevitable in real-world scenarios and further demonstrate the necessity of prospective evaluation systems such as this to understand how the model will perform in the clinical setting before adoption efforts and its widespread use.

In addition to demonstrating the feasibility of integrating a custom ML model into a live clinical system and sharing how we built this system in our Methods section, we also outline insights for future studies to consider as they build upon this work.

First, idiosyncrasies of the healthcare environment may force adaptable approaches. For example, we found that a nurse or supporting clinician typically placed the Heme-STAMP order, rather than the attending physicians, whose predictions we were evaluating. This made instantaneous human data collection impractical, requiring a creative approach to still gather real-time prospective clinician estimates. We used a silent EHR alert that triggered FHIR-based API calls and a semi-automated email system to gather physician estimates of freshly placed orders. This created a short delay between test ordering and acquisition of physician estimates, but was still well before actual test results were available. While this approach could inadvertently exclude some cases, our prospective cohort remained representative of the comprehensive list of Heme-STAMP orders used for model training (Supplementary Table 3).

Second, despite the model’s strengths, we found participating physicians to be reluctant to adjust their estimates when shown model estimates. Thus, in addition to developing a robust model, an equally important task lies in user design, namely designing *how* the model estimates are shown, such that they can most effectively be combined with physician intuition. In our study, we conducted secondary analyses and found that averaging physician and model prediction probabilities was more effective than simply showing the physician the model estimate or averaging their estimates with a second opinion. The former averaged approach maintained strong discriminatory power (AUC score) while improving calibration. Other prospective evaluation studies may consider incorporating a design that first prompts physicians for their predicted probabilities, averages this with the model, and then shows the combined average, rather than the more common design that simply shows the model estimates. This approach, along with information on how the averaged combination maintains performance while improving calibration, may provide a cost-effective and simple way of improving model acceptance into clinical workflow.

Third, although the model was able to perform comparably to an expert physician without tertiary information, it may be valuable to build pipelines to import such data as well. We found that tertiary information sources, such as the Care Everywhere health information exchange, contained important information such as reports of previous mutations or known hematologic diagnoses. Many of the expert physicians in our study used information from these sources to guide their decision-making. Thus, while such information is not necessary for comparable model performance, it certainly may further improve model performance.

Finally, close collaboration with clinicians is essential in developing an effective ML-based clinical decision support tool. We worked with our multidisciplinary team of front-line clinical co-authors (M.S., R.B., D.I., W.S., J.S.O., D.G., and J.H.C.) to ensure the tool’s relevance to clinical decision-making. This study applies decision analysis^8^, using thresholding to distinguish when further testing is needed. If outcomes were entirely unpredictable (AUROC = 0.5), testing would be required for all cases. However, with an AUROC of 0.77, the model demonstrates predictive capability, supporting threshold-based decision-making. Discussions with clinical co-authors highlighted that thresholding strong negative predictions was more clear and actionable than positive cases. At NPVs of 0.9 and 0.95, the model outperformed physicians in true negative rate (TNR). Prior research^15^ indicates that physicians are willing to change decisions at a model certainty level of 0.8, suggesting that at this threshold, our model reliably identifies negative outcomes, potentially negating the need for testing in those cases. The utility of a positive Heme-STAMP result is more complex, as the panel covers hundreds of genes, many with uncertain significance. The subjective nature of determining “usefulness” makes it an impractical study outcome. In contrast, a negative prediction is often actionable. Though the model does not definitively determine whether to order Heme-STAMP testing, it provides a crucial pre-test probability. By working closely with clinicians, we ensured that the model’s prediction granularity (exact percentages vs. stratified categories), certainty level, and utility aligned with physician needs, enhancing its clinical applicability.

A limitation of the study is that the model may have been based on prior clinical behavior rather than contributing a fresh perspective. Allopurinol prescription, which is common in leukemia and lymphoma care to prevent the effects of tumor lysis syndrome, was among the most predictive factors, suggesting that the model was sometimes “looking over the clinician’s shoulders” rather than identifying new information^16^. The extent to which the model learns from such *processes* of medicine may also limit its portability in different clinical environments, such as settings where allopurinol orders are associated with other diseases. This suggests that deliberate testing in each intended clinical setting is warranted.

A nuanced analysis of estimate alignment showed that while the majority of cases (>70%) demonstrated directional alignment between the model and the ordering physician, a minority of cases had discrepancy and were further investigated through manual review (Supplementary Table 6). Some differences were due to physician overconfidence about a diagnosis or overlooking elements of the patient’s history. Additional differences were due to the model not incorporating the disease timeline into the prediction, since mutations are often cleared during disease remission. Other discrepant predictions were due to technical issues in sequencing, such as a case having a known blood cancer mutation that was at too low a level to be detected.

Another risk of ML model training is inadvertently learning undesirable bias related to patient race, age, and sex^17^, especially since some hematologic parameters vary with self-reported race. For example, the Duffy-null phenotype is evident in a large proportion of Black individuals, which results in normal neutrophil counts below the range defined among Duffy-positive individuals^18^. Referral bias and access to specialty care may also adversely influence ML predictions. Although self-reported race was a salient feature in the SHAP analysis, the effect on prediction probabilities was mixed (both positive and negative contributors), not reflecting a consistent statistical bias in either direction. Age and sex were identified in the SHAP analysis as significant predictors of mutations, consistent with previous studies on hematologic malignancies; mutations are known to accumulate in blood as people age^13^, and many blood diseases are more common in men than in women^19,20^. Interestingly, we noted relatively lower performance of both clinician predictions and our model’s predictions in the older half of the patient population (Supplementary Table 4, Supplementary Fig. 5). This suggests that the model’s index on older age as a proxy to a positive test may be overly confident, leading to false positives. Future studies that are powered to study these specific subgroups may improve predictions with a larger sample size or by training models on specific age groups.

Another limitation stems from acknowledging that as our knowledge about cancer mutations increases, any model will have to be adjusted. We assessed all variants that were defined as pathogenic and likely pathogenic as a positive result, but some low-level “pathogenic” mutations (e.g., select mutations in *TET2*) have unclear clinical significance^13^. Furthermore, variants of unclear significance that were considered “negative” in this study may later be reclassified as pathogenic.

In conclusion, we found that machine learning models trained on readily available structured EHR data can predict the results of a complex NGS test with discriminatory power comparable to expert hematologists, despite the model having less access to data than their physician counterparts. Furthermore, combining the model with physician input maintains strong discriminatory power (AUC performance) that is on par with expert physicians while also improving calibration (Brier Score, calibration plots). Lastly, despite the growing popularity of machine learning in clinical environments, many are not able to move beyond the piloting stage because of challenges with integrating into a live clinical environment. This study demonstrates that it is feasible to integrate a custom machine learning model into a real-time clinical environment and exemplifies how to do so using FHIR-based Epic API calls, the mechanism of an EHR alert, and Microsoft Azure’s cloud computing platform.

## Methods

### Study design

**Fig. 4:**
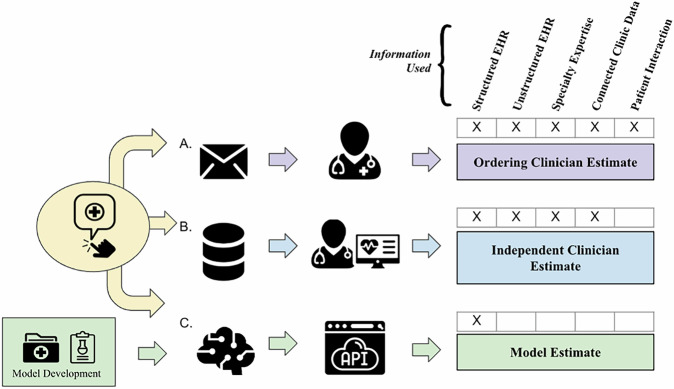
Study arms: machine learning model, ordering clinician, and independent clinician. When a Heme-STAMP order is placed by the participating hematologists, a silent EHR alert is triggered, initiating the three study arms to obtain estimates of pathogenicity. **Arm A:** Order information is used to generate an email (Supplementary Fig. 1) that includes the order date, patient medical record number, name, latest white blood cell count, and latest hemoglobin levels - values that remind the hematologists of relevant patient information. Orders placed over one to two week periods are batched into a consolidated query email to reduce email burden. This batching still protects the integrity of prospective predictions since the turnaround time for Heme-STAMP is around three weeks. These emails are sent to the respective ordering hematologists to obtain their estimates of a pathogenic result in buckets of <10%, 10–30%, 30–50%, 50–70%, 70–90%, and >90%. **Arm B:** Order information is saved in a secure database server. These cases are later reviewed by an independent hematologist who uses chart review up to the date of the order to provide their estimate. **Arm C:** The automated ML model integrated into the live EHR uses data directly from Epic Chronicles, the EHR’s transactional database that stores up-to-date patient data, to query structured patient information (e.g., demographics, diagnosis codes, prior lab results, and medications) needed to calculate the probability of a pathogenic test result. We developed a DEPLOYR^14^ framework that uses fast healthcare interoperability resources (FHIR)^28^-based Epic application programming interfaces (API) calls to pull live feature data, with the appropriate mappings to training feature data, in order to enable this real-time prediction.

Figure 4 diagrams the overall study design, with multiple arms predicting whether Heme-STAMP NGS tests would result in finding *any* pathogenic variants (positive result) or no known pathogenic variants (negative result). Arm B in Fig. 4 represents gathering retrospective hematologist estimates based on the chart review as a baseline of comparison. In addition to these retrospective estimates, we *prospectively* generated model estimates and gathered the ordering physicians’ real-time estimates while they awaited results. This was enabled by using our DEPLOYR^14^ framework, which uses serverless Azure functions, FHIR-based Epic API calls, and best practice alert web service triggers to allow for custom machine learning model integration into live EHR workflows. These real-time physician and model prediction pathways can be seen in Arm A and C of Fig. 4, respectively. The details of how this model was trained can be found in the Model Development section below. We completed an a priori power analysis to pre-specify a sample size of 100 prospective cases. This was based on preliminary data^9^ of machine learning model performance and deeming a difference in AUROC performance between model vs. human predictions of 0.80 vs. 0.60 to be clinically significant. The sample size was then estimated with the *power.roc.test* function of the *pROC* R package^21^ with an alpha of 0.05 and power of 0.80.

### Setting and participants

Patients were seen by a hematologist at an academic medical center, where the physician ordered a Heme-STAMP test as part of routine clinical care from June 7, 2022 to March 5, 2023. The three participating hematologists who placed Heme-STAMP orders see patients with all blood cancers but focus on particular disorders – Dr. Shomali on myeloproliferative neoplasms and myelodysplastic syndromes, Dr. Iberri on plasma-cell disorders, and Dr. Brar on challenging diagnoses and complement disorders. The Heme-STAMP test could be ordered at any time in the patient’s clinical course, including in those already known to have blood cancer mutations. These three ordering hematologists provided estimates of the likelihood of a positive (pathogenic or likely pathogenic) result on their Heme-STAMP orders. An independent board-certified hematologist, Dr. Schwede, also provided likelihood estimates on orders placed by these hematologists. The study was approved by the Stanford Institutional Review Board (IRB-65247). We complied with all relevant ethical regulations and obtained informed consent from all participants.

### Labels

Heme-STAMP interrogates over two hundred genes (Supplementary Table 1) selected for their therapeutic, diagnostic, or prognostic relevance in hematolymphoid malignancies for a number of variant types (single-nucleotide variants, small insertions and deletions, genomic rearrangements). The primary test result is a list of DNA variants detected in the specimen. These variants are further classified based on expert molecular pathologist review of existing literature and evidence, into pathogenic (disease-causing), likely pathogenic, benign (not disease-causing), or likely benign. Variants that cannot be confidently classified into one of these groups are assigned the status Variant of Unknown Significance (VUS).

In deciding whether to develop pathogenicity predictions for each gene or for the overall panel (namely, determining if at least one pathogenic variant was found), we reviewed with multiple practicing physicians who indicated the level of granularity given by gene-level predictions to be unnecessarily complicated information to interpret and thus preferred a user-interface design that used the latter, panel-level approach.

Figure 1 illustrates the respective Heme-STAMP test results in binary labels: *positive* if at least one pathogenic or likely pathogenic variant was identified, and *negative* otherwise.

### Model development

The Stanford Research Repository (STARR)^22^ is a clinical data warehouse that stores a copy of EHR data from Stanford Health Care and the Stanford Children’s Hospital. We used EHR data from this repository to train a random forest classifier^23^ with 1000 estimators (otherwise default parameters) to predict Heme-STAMP results. In prior work^9^, we tested multiple models including logistic regression, random forest, and XGBoost. The random forest and XGBoost models outperformed the linear model but had minimal performance difference between each other (Supplementary Table 2). Since this model would be deployed in the live EHR environment, we broke the tie between random forest and XGBoost based on considerations related to the ease of deployment and ultimately selected the random forest model. Hyperparameters were selected based on a grid search (Sklearn.GridSearchCV) across the number of estimators (100, 500, 1000), maximum depth (None, 80, 90, 100, 110), minimum number of samples required to split (2, 8, 10, 12), and the minimum number of samples required to be at a leaf node (1, 3, 4, 5). The model was fairly robust to the changing parameters, and the optimized hyperparameters did not yield statistically significant improvement from the model with default hyperparameters. The best Random Forest model optimized across all the parameters listed above was RandomForestClassifier (n_estimator = 1000, min_samples_split = 10, max_depth = 90) with an AUROC of 0.77 [0.74, 0.80]. The best Random Forest model optimized across just the n_estimator hyperparameter was RandomForestClassifier(n_estimator = 1000) with an AUROC of 0.76 [0.72, 0.79]. The default Random Forest model had an AUROC of 0.76 [0.72, 0.79].

The structured health record data used to construct predictive features includes demographics (sex, age, race), medications, and ICD10 diagnoses recorded by the time of the Heme-STAMP order, and labs resulting within a 14-day lookback window from the index time of a prediction. Numeric features were binned into five buckets based on their training set distributions, as previously described^24^, and labs were analyzed as counts in each such bucket. Data elements were extracted for 3,472 Heme-STAMP orders placed between May 2018 and September 2021 from 3220 unique patients. To train and validate the model retrospectively, we used a time-based train/test split^25^. We mapped features from STARR to data elements directly accessible through real-time Epic API calls to ensure consistency of the retrospectively trained model with real-time prospective prediction implementation in a production environment.

### Evaluation Metrics

Each study arm was evaluated based on how well the method could predict the Heme-STAMP outcome labels based on the following evaluation metrics (with confidence intervals obtained through bootstrapping):

1. AUROC (c-statistic): Area Under the Receiver Operating Characteristic curve summarizes overall discrimination performance, reflecting the tradeoff between sensitivity vs. specificity.
2. AP: Average Precision reflects the Area Under the Precision-Recall curve, which captures the tradeoff between precision (positive predictive value) and recall (sensitivity).
3. Brier Score: Measures the accuracy of a predicted probability.
4. Calibration: Measures how accurately the predicted probability matches the observed probability.
5. NPV: Negative Predictive Value is the proportion of negative predictions that were true negatives.
6. TNR: True Negative Rate or Specificity is the proportion of negative cases that were predicted to be negative. We focused on TNR values achievable at high NPV values of 0.90 and 0.95. A TNR value of X% at an NPV value of 0.90 would indicate that a method correctly classified X% of the negative cases while ensuring that 90% of its negative predictions were accurate.

The SHAP (SHapley Additive exPlanations)^26^ framework was used to analyze the model features important for prediction. SHAP facilitates the interpretation of complex prediction models by summing the contributions of each feature towards predictive performance. The base value for prediction in a classifier model is the prevalence of the positive class, while the contribution of each feature is represented by the difference in prediction values (Δ) attributable to including the feature. Because models (such as the random forest models used here) may have non-linear and non-independent features, a feature’s contribution may differ depending on the order in which features are added to the model. SHAP values for each feature are thus calculated by averaging the Δ value across all possible feature addition orderings.

To assess the correlation between different prediction approaches, we calculated Kendall’s Tau-b Coefficient^27^ values between each pair-wise combination of approaches. These non-parametric tests measure rank correlation between predictions. Prediction probabilities from the machine learning model were binned into rank-ordered groups of >90%, 70–90%, 50–70%, 30–50%, 10–30%, and <10% to match the ordinal ranking scheme of the physician predictions. We also calculated Spearman Rank Correlation Coefficient values to verify trends seen with Kendall’s, although Kendall’s was the primary statistic because it is a more robust^3^ and conservative^4^ correlation measurement. Correlation coefficient values can range from −1 to +1, with the sign reflecting the nature of the correlation and the magnitude reflecting the strength of the correlation, where -1 and +1 reflect perfect correlation, and 0 reflects no correlation.

### Technical framework for real-time model evaluation

We utilized the DEPLOYR^14^ framework to integrate our machine learning model into the live medical record of an academic medical center and generate real-time predictions on lab orders as they were placed. Fast healthcare interoperability resources (FHIR)-based Epic application programming interface (API) calls were used to pull feature data (e.g., demographics, diagnosis codes, prior lab results, and medications) directly from Epic Chronicles, the EHR’s transactional database that stores real-time patient data, to generate these predictions. The API function documentation details were found on Epic AppOrchard via vendorservices.epic.com. Examples of these function calls can be found in the GitHub repository (see Code Availability). Features included in the feature vector were mapped to the appropriate function call found in the documentation in vendorservices.epic.com. An example of some of these data mappings can be found in Supplementary Fig. 2. These API calls were included in a python script that was triggered by an EHR alert (a Best Practice Advisory in the Epic system) that ran silently (not shown to the user) when an order was placed to generate the model estimates. The model and the Python script were stored on Microsoft Azure’s computing platform, and the model estimates were saved on Azure Cosmos DB, a serverless database.

### Technical framework for prospective physician evaluation

We developed a semi-automated emailing system to gather prospective estimates from physicians as they placed lab orders. By leveraging the triggering mechanism of an EHR alert (Best Practice Advisory in the Epic system) but not actually displaying the alert to any user, we were able to capture order information as orders were placed on Epic. This information was saved to a secure database and then used alongside FHIR-based Epic API calls to extract relevant information regarding the order. All of this information was organized in a table format and sent to the ordering physician in an auto-templated email. The physician could then respond with their estimate of pathogenicity for each of the Heme-STAMP tests they had ordered. This response was then processed automatically and saved in a secure database. See Supplementary Fig. 1 for a sample email.

### Ensemble and “Second Opinion” averaging

To understand the potential of combining different approaches (e.g., human + computer), we averaged prediction probability estimates from each of the three arms to produce ensemble estimates. For example, given a Heme-STAMP order, we took the arithmetic mean of the machine learning model’s probability estimate for finding a pathogenic result and the ordering physician’s estimate to produce a combined “model + ordering clinician” estimate. This approach was used to generate model + ordering clinician, model + independent clinician, ordering clinician + independent clinician, and model + ordering clinician + independent clinician ensemble estimates.

## Supplementary information

Supplementary Material

## Data Availability

The deidentified feature data, labels, and prediction probabilities are available at 10.5061/dryad.nzs7h450b.

## Code availability

The underlying code for this study and training/validation datasets is available at https://github.com/HealthRex/CDSS/tree/master/scripts/Heme-Stamp.
