# Supplementary material for: Real time machine learning prediction of next generation sequencing test results in live clinical settings
Source: NPJ Digit Med. 2025 Aug 19;8:533. doi: 10.1038/s41746-025-01816-7 (PMC12365101; doi:10.1038/s41746-025-01816-7)
Supplement: Supplementary file 1 — Supplementary Material [file 41746_2025_1816_MOESM1_ESM.pdf]

Supplementary Information

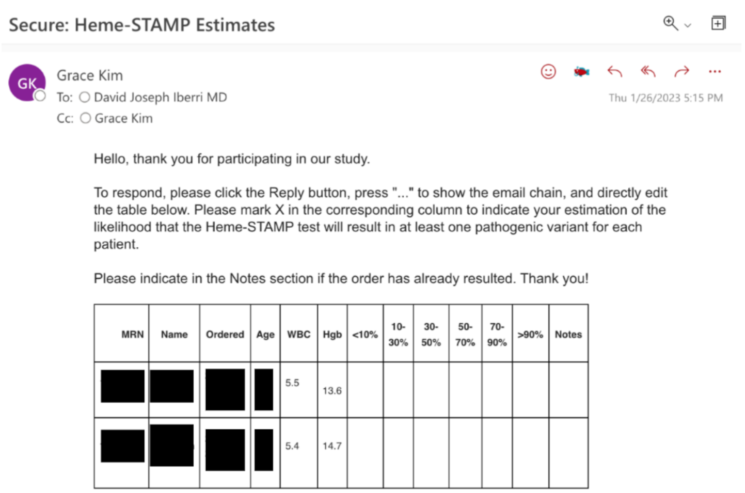
Secure: Heme-STAMP Estimates

Grace Kim
To: David Joseph Iberri MD
Cc: Grace Kim
Thu 1/26/2023 5:15 PM

Hello, thank you for participating in our study.

To respond, please click the Reply button, press "..." to show the email chain, and directly edit the table below. Please mark X in the corresponding column to indicate your estimation of the likelihood that the Heme-STAMP test will result in at least one pathogenic variant for each patient.

Please indicate in the Notes section if the order has already resulted. Thank you!

MRN	Name	Ordered	Age	WBC	Hgb	<10%	10-30%	30-50%	50-70%	70-90%	>90%	Notes
[redacted]	[redacted]	[redacted]	[redacted]	5.5	13.6							
[redacted]	[redacted]	[redacted]	[redacted]	5.4	14.7							

Supplementary Figure 1 | Sample Email Gathering Prospective Estimates from Ordering Physicians. Sample email sent to ordering clinicians querying for prospective estimates on recent Heme-STAMP orders. Additional information on the order such as MRN, patient name, date ordered, patient age, recent WBC count, and recent Hgb count were included as well. Physicians responded by marking an X on the column (<10%, 10-30%, 30-50%, 50-70%, 70-90%, >90%) that best corresponded with their estimated probability of a positive Heme-STAMP lab result.

ABL1	CD28	ETV6	IGHM	MYD88	POT1	STIL
ABL2	CD58	EZH2	IKZF1	MYH11	PPM1D	STX11
AKT1	CD79A	FAS	IKZF2	NF1	PRDM1	SYK
ALK	CD79B	FBXW7	IKZF3	NFKB2	PTEN	TAL1
ANKRD26	CD83	FGFR1	IL2RB	NFKBIE	PTPN11	TBL1XR1
APLNR	CDKN2A	FGFR3	IL7R	NOTCH1	RAD21	TCF3
APLNR	CDKN2B	FIP1L1	IRF4	NOTCH2	RARA	TERT
ASXL1	CDKN2C	FLT3	IRF8	NPM1	RB1	TET2
ATM	CEBPA	FOXO1	ITCH	NR3C1	RHOA	TMEM30A
B2M	CEP72	FYN	ITK	NRAS	RPS15	TNFAIP3
BCL10	CIITA	GATA1	JAK1	NSD2	RRAGC	TNFRSF14
BCL2	CKS1B	GATA2	JAK2	NT5C2	RUNX1	TNFRSF1B
BCL6	CREBBP	GATA3	JAK3	NUDT15	S1PR2	TP53
BCOR	CRLF2	GLIS2	KDM6A	NUP214	SET	TPMT
BCR	CSF1R	GNA13	KIT	P2RY8	SETBP1	TRAF2
BIRC3	CSF3R	GNAS	KLF2	PAX5	SETD2	TYK2
BRAF	CSNK1A1	GNB1	KLHL6	PDCD1	SF3B1	U2AF1
BTK	CTLA4	HAVCR2	KMT2A	PDCD1LG2	SGK1	UBA1
CALR	CUX1	HNRNPK	KRAS	PDGFRA	SH2B3	VAV1
CARD11	CXCR4	HRAS	LAG3	PDGFRB	SMARCB1	WT1
CARMIL2	DDX3X	ICOS	LCK	PHF6	SMC1A	XPO1
CBFA2T3	DDX41	ID3	LYN	PIGA	SMC3	ZNF384
CBFB	DEK	IDH1	MALT1	PIK3CA	SOCS1	ZRSR2
CBL	DNMT3A	IDH2	MAP2K1	PIK3CD	SPOCD1	
CBLB	EBF1	IGHA1	MAPK1	PIM1	SRSF2	
CCND1	EGR1	IGHA2	MEF2B	PLCB1	STAG2	
CCND2	EP300	IGHG1	MEF2D	PLCG1	STAT1	

CCND3	EPOR	IGHG2	MPL	PLCG2	STAT3	
CCR4	ERG	IGHG3	MTOR	PML	STAT5B	
CD274	ETNK1	IGHG4	MYC	POLE	STAT6	

Supplementary Table 1 | Heme-STAMP Panel. Genes assayed in Heme-STAMP panel as of April 2022.

Model	AUROC
Logistic Regression	0.66 [0.63, 0.69]
Random Forest	0.72 [0.70, 0.74]
XGBoost	0.74 [0.72, 0.76]

Supplementary Table 2 | Model AUROC Comparison. AUROC of logistic regression, random forest, and XGBoost models trained and validated on retrospective Heme-STAMP data in prior work[8] to determine which model to build upon for expansion work described in this manuscript.

Description	Category	Retrospective Cohort (Train/Validation)	Prospective Cohort (Test)
n	Total	3,472	101
Label, n (%)	Pathogenic	2116 (61)	66 (65)
	Non-Pathogenic	1356 (39)	35 (35)
Order Type, n (%)	First order	2787 (80)	75 (74)
	Repeat order	685 (20)	26 (26)
Sex, n (%)	Female	1549 (45)	53 (52)
	Male	1922 (55)	48 (48)
Race, n (%)	White	2000 (58)	66 (65)
	Other	556 (16)	16 (16)
	Asian	526 (15)	8 (8)

	Unknown	243 (7)	6 (6)
	Black	91 (3)	3 (3)
	Pacific Islander	49 (1)	2 (2)
Age	Median (IQR)	64 (23)	67 (21)
Specimen Type, n (%)	Peripheral Blood	1460 (42)	54 (53)
	Bone Marrow	2012 (58)	47 (47)
White Blood Cell Count (K/uL), n (%)	1st quintile: <1.2	1132 (21)	28 (23)
	2nd quintile: 1.2-3.2	1063 (20)	16 (13)
	3rd quintile: 3.2-5.9	1038 (19)	26 (21)
	4th quintile: 5.9-12.6	1076 (20)	34 (28)
	5th quintile: >12.6	1073 (20)	18 (15)
Platelet Count (K/uL), n (%)	1st quintile: <23	1125 (21)	16 (13)
	2nd quintile: 23-51	1040 (19)	30 (25)
	3rd quintile: 51-109	1077 (20)	29 (24)
	4th quintile: 109-212	1078 (20)	31 (25)
	5th quintile: >212	1076 (20)	16 (13)

Supplementary Table 3 | Patient Demographics, Lab Details, and Order Details of Train and Test Cohorts. Demographics of patients along with labs and order details in train/validation and test sets. The train/validation dataset consists of retrospective cases from May 2018 - September 2021 and the test dataset consists of cases ordered between June 2022 - March 2023 for which estimates were prospectively obtained. Patients were mostly white and over 65 years old, and most specimens were the first Heme-STAMP order for each patient. The case characteristics of the prospective dataset were generally representative of the retrospective dataset.

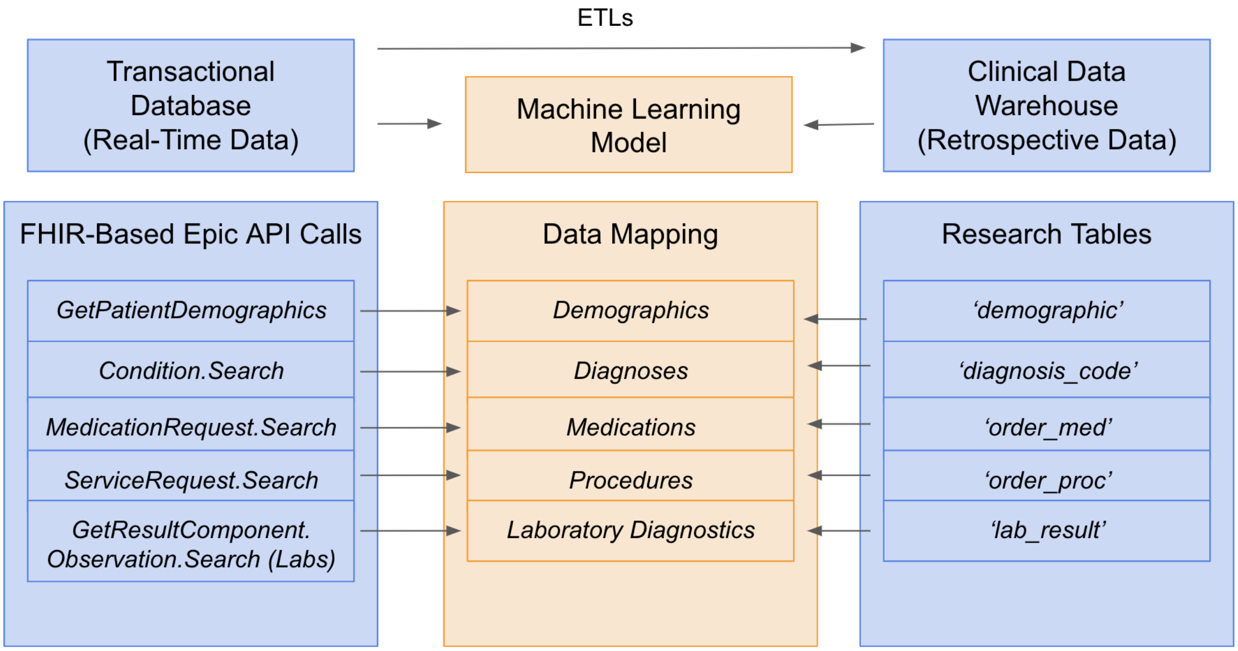

Supplementary Figure 2 | Mapping Retrospective Data and Real-Time Data to Model Features. Retrospective data retrieved from the clinical data warehouse and real-time data pulled from the EHR transactional data through FHIR-based Epic API calls must undergo data processing to map to features included in the machine learning model.

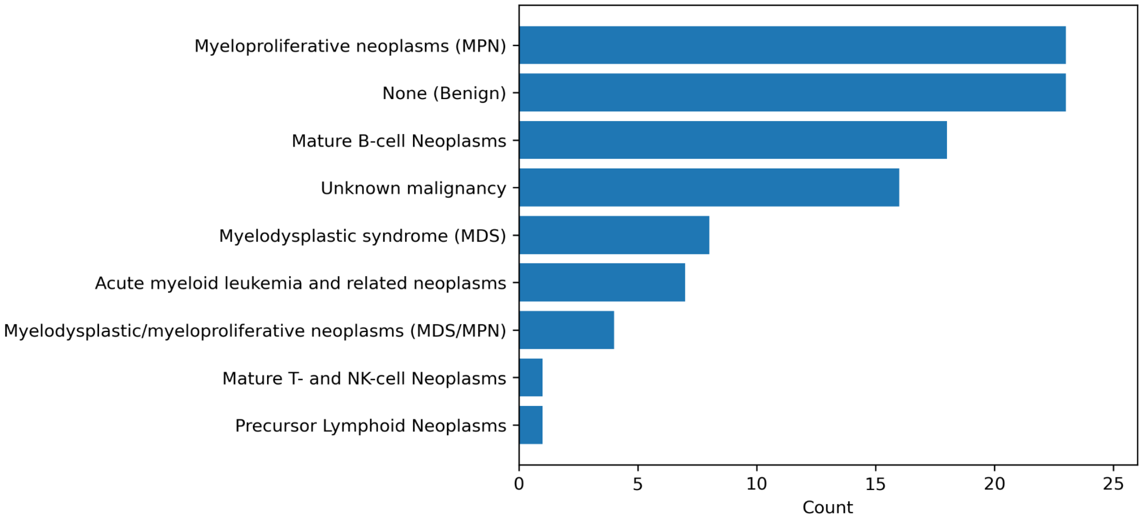

Supplementary Figure 3 | Most Common Diagnoses Among Patients with Heme-STAMP Lab Orders Hematological diagnoses of patients included in the Prospective Cohort (test set) were obtained through manual review by the molecular pathologist and/or hematologist. The most common categories of these diagnoses are shown above.

Cohort			All	Bone Marrow	Peripheral Blood	Age < 67	Age ≥ 67
n (%)			101 (100)	47 (47)	54 (53)	50 (50)	51 (50)
Model	Ordering Clinician	Indep. Clinician	AUROC Scores [95% CI]				
+	-	-	0.77 [0.66, 0.87]	0.76 [0.59, 0.91]	0.80 [0.65, 0.91]	0.81 [0.66, 0.92]	0.58 [0.37, 0.80]
-	+	-	0.78 [0.68, 0.86]	0.75 [0.59, 0.89]	0.79 [0.67, 0.90]	0.84 [0.73, 0.94]	0.63 [0.44, 0.81]
-	-	+	0.72 [0.62, 0.81]	0.72 [0.56, 0.86]	0.76 [0.60, 0.88]	0.77 [0.62, 0.89]	0.57 [0.38, 0.76]
+	+	-	0.83 [0.73, 0.91]	0.78 [0.61, 0.92]	0.85 [0.74, 0.95]	0.90 [0.80, 0.98]	0.61 [0.37, 0.84]
+	-	+	0.79 [0.69, 0.87]	0.78 [0.62, 0.92]	0.83 [0.70, 0.94]	0.84 [0.73, 0.94]	0.61 [0.40, 0.81]
-	+	+	0.80 [0.70, 0.88]	0.78 [0.62, 0.90]	0.83 [0.72, 0.93]	0.87 [0.77, 0.96]	0.63 [0.43, 0.81]
+	+	+	0.82 [0.73, 0.90]	0.79 [0.64, 0.93]	0.85 [0.72, 0.95]	0.90 [0.79, 0.98]	0.63 [0.42, 0.82]
Model	Ordering Clinician	Indep. Clinician	AP Scores [95% CI]				
+	-	-	0.84 [0.74, 0.93]	0.88 [0.76, 0.97]	0.83 [0.68, 0.94]	0.80 [0.63, 0.93]	0.85 [0.73, 0.95]
-	+	-	0.83 [0.73, 0.91]	0.87 [0.75, 0.96]	0.79 [0.66, 0.91]	0.79 [0.64, 0.93]	0.86 [0.75, 0.95]
-	-	+	0.80 [0.69, 0.90]	0.87 [0.76, 0.95]	0.76 [0.60, 0.88]	0.75 [0.59, 0.90]	0.84 [0.70, 0.95]
+	+	-	0.88 [0.79, 0.95]	0.89 [0.76, 0.98]	0.87 [0.73, 0.97]	0.90 [0.77, 0.98]	0.86 [0.74, 0.97]
+	-	+	0.86 [0.77, 0.94]	0.90 [0.80, 0.98]	0.85 [0.72, 0.96]	0.86 [0.72, 0.95]	0.87 [0.75, 0.96]
-	+	+	0.87 [0.78, 0.93]	0.91 [0.82, 0.97]	0.84 [0.70, 0.95]	0.89 [0.79, 0.97]	0.87 [0.74, 0.96]
+	+	+	0.88 [0.79, 0.95]	0.91 [0.81, 0.98]	0.87 [0.73, 0.97]	0.93 [0.83, 0.98]	0.88 [0.75, 0.97]

Supplementary Table 4 | AUROC and AP Scores. Area Under the Receiver Operating Characteristic (AUROC / c-statistic) and Average Precision (AP) scores of different approaches to predicting whether a Heme-STAMP next-generation sequencing test will yield any pathogenic gene mutation results. Scores reflect performance on a prospectively collected set of cases with predictions made by the model, the ordering clinician, an independent clinician, or ensemble combinations of those three approaches. Performance on subsets of sample type (bone marrow

or peripheral blood) and age (less than 67 or greater than 67) are included as well. The median age of the prospective cohort, 67, was used as the age threshold.

Predictor Pair	Kendall Tau-b Coefficient	Spearman Coefficient
Model - Ordering Clinician	0.34 ($p < 10^{-4}$*)	0.41 ($p < 10^{-4}$*)
Model - Independent Clinician	0.32 ($p < 10^{-4}$*)	0.42 ($p < 10^{-4}$*)
Ordering Clinician - Independent Clinician	0.45 ($p < 10^{-4}$*)	0.55 ($p < 10^{-8}$*)

Supplementary Table 5 | Kendall Tau-b and Spearman Correlation Coefficients for Each Predictor Pair. Correlation coefficient values can range from -1 to +1 with the sign reflecting the nature of correlation and the magnitude reflecting the strength of correlation. -1 and +1 reflect perfect correlation (in opposite or similar directions, respectively) and 0 reflects no correlation. The statistically significant positive correlation between the model and clinicians indicates that the model's predictions are similar to those of the physicians. However, the modest correlation suggests that the model is basing predictions on unique factors rather than simply duplicating the physician. *$p<0.05$

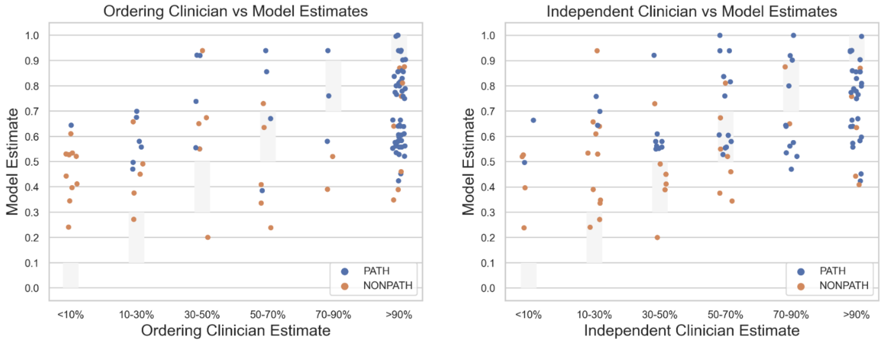

Supplementary Figure 4 | Physician vs Model Scatter Plots. Model estimates of a pathogenic/positive outcome (continuous: [0,1]) were plotted against physician estimates (categorical: 10%, 10-30%, 30-50%, 50-70%, 70-90%, >90%). The gray bars provide a frame of reference of the physician estimation categories when viewing the continuous model estimates. The colors indicate the ground truth outcome of the lab test (pathogenic or nonpathogenic).

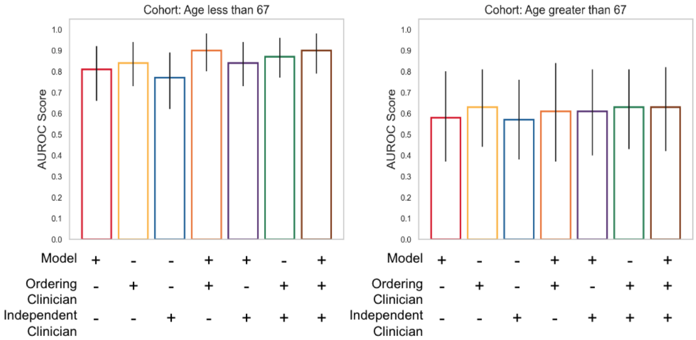

Supplementary Figure 5 | AUROC Score of Different Predictor Approaches Among Patients of Age Greater or Less Than 67. AUROC score of predictions made by the model, the ordering clinician, independent clinician, and ensemble combinations of those three approaches on cases with patients of age less than 67 (positive prevalence: 0.5

= 25/50) and age greater than or equal to 67 (positive prevalence: 0.8 = 41/51). The median age of the total cohort was 67.

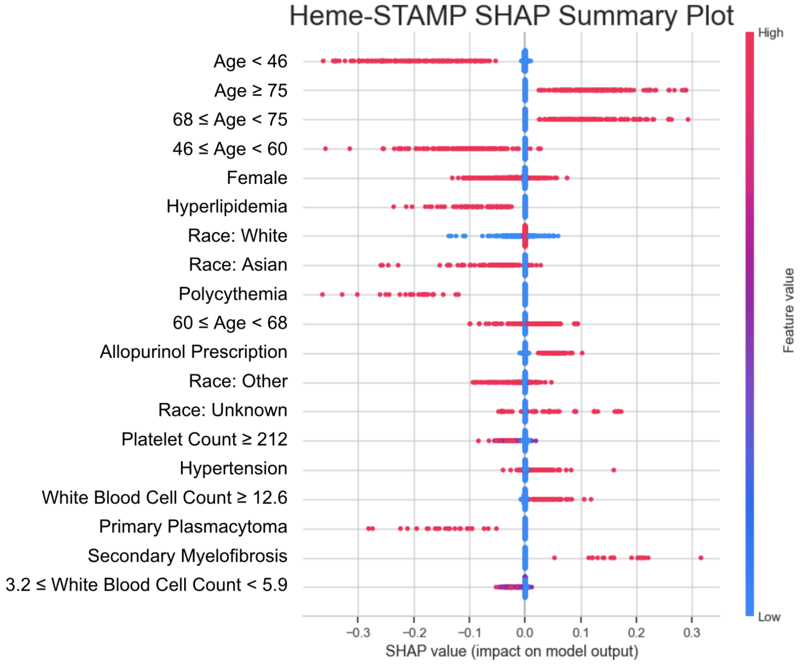

Supplementary Figure 6 | SHAP Feature Importance Plot. This beeswarm plot depicts the relative importance of the model's features for its predictions using each variable's SHAP values. The top features, ordered by importance are shown. Here, importance is based on the mean absolute SHAP value across the dataset. The SHAP value is plotted for each feature, with each dot corresponding to a Heme-STAMP test (thus 101 dots per line). Red color indicates that the feature is present, and blue indicates absence, except laboratory value buckets which are represented on a spectrum as counts (i.e., number of times a patient has had a laboratory result in that bucket range). The SHAP value plotted on the x-axis indicates whether the feature value increased (right side) or decreased (left side) the estimated probability of a positive Heme-STAMP for that specific test. For example, when the feature "Age ≥ 75" is present (red), the SHAP values are positive (right side), indicating that "Age ≥ 75" corresponds to a higher predicted probability of a positive Heme-STAMP test result. In the "Platelet Count ≥ 212" row, the color spectrum represents the number of Platelet tests for which the count was ≥ 212. Patients with dots colored closer to red had a higher number of platelet tests that resulted in Platelet count ≥ 212. It appears that having more of these high Platelet count cases usually (but not always) indicated a lower probability of a positive Heme-STAMP.

Class	Model Estimate	Ordering Clinician Estimate	Independent Clinician Estimate	Diagnosis	Specimen Type	Age/Sex	Reason for discrepancy
NONPATH	0.93910873	30-50%	10-30%	Hematologic and Lymphatic Neoplasm - Mature B-cell Neoplasms - Hairy cell leukemia	Peripheral blood (PB)	69M	Disease in remission
PATH	0.92114994	30-50%	30-50%	Hematologic and Lymphatic Neoplasm - Mature B-cell Neoplasms - Chronic lymphocytic leukemia/small lymphocytic lymphoma	Peripheral blood (PB)	81M	Unclear diagnosis

PATH	0.42368866	>90%	>90%	None (Benign)	Peripheral blood (PB)	29M	Known prior mutation
NONPATH	0.61005727	<10%	10-30%	None (Benign)	Peripheral blood (PB)	65M	Known negative mutation
NONPATH	0.38890818	>90%	30-50%	None (Benign)	Peripheral blood (PB)	63F	Error in clinician judgment
NONPATH	0.44257143	<10%	>90%	Hematologic and Lymphatic Neoplasm	Peripheral blood (PB)	63M	Error in clinician judgment
NONPATH	0.40887166	50-70%	>90%	Hematologic and Lymphatic Neoplasm	Peripheral blood (PB)	40M	Known prior mutation, but Heme-STAMP not sufficiently sensitive
PATH	0.75828403	>90%	10-30%	None (Benign)	Bone marrow (BM)	83M	Error in clinician judgment

Supplementary Table 6 | Cases with Discrepant Predictions. While most cases had alignment between model and physician predictions, the table shows the few cases that had highly discrepant predictions (absolute prediction difference > 50%) between the model and either the ordering clinician or the independent clinician.
